# Editorial: Pet obesity: health impacts and innovations in weight management

**DOI:** 10.3389/fvets.2026.1822501

**Published:** 2026-05-19

**Authors:** Andressa Rodrigues Amaral, Pedro Henrique Marchi, Mariana Fragoso Rentas, Thiago Vendramini

**Affiliations:** 1 School of Veterinary Medicine and Animal Science, University of São Paulo, São Paulo, Brazil; 2 Centro Universitário UNINTA, Sobral, Ceará, Brazil

**Keywords:** cats, diagnostic, dogs, lifespan, obesity, risk factors, treatment

## Prevention, diagnostic, and treatment

Effective prevention begins with the identification of risks. The study by Bjørnvad et al. investigated risk factors in Danish cats, revealing that indoor confinement increases the probability of domestic shorthaired cats becoming heavy or obese as early as 1 year of age. An important finding of this research was the association between feline obesity and a stronger emotional attachment from the owner, suggesting that human perception and family lifestyle play a determining role in excessive energy intake.

Complementing this perspective, the longitudinal study by Pye et al. followed 209 cats for up to 7 years, demonstrating that muscle mass loss begins even before middle age (7 years). In contrast, the loss of body weight and body condition score typically becomes evident only after 10 years of age. These data underscore the vital importance of monitoring muscle condition early to differentiate healthy aging from changes associated with chronic diseases.

While the body condition score (BCS) remains the standard clinical tool, this Topic presents advances in metabolic biomarkers that allow for a more refined diagnosis of “obesity.” Kobayashi et al. proposed new diagnostic criteria for cats using a highly sensitive serum amyloid A (SAA) assay. A diagnosis of pathological obesity was established for cats with a BCS ≥ 7/9 exhibiting two or more of the following: hyperlipidemia (TG ≥ 180 mg/100 mL), elevated ALT activities (≥ 80 U/L), and high SAA values (≥ 4.0 mg/L), enabling clinical interventions before severe organ damage occurs.

In dogs, research by Gomez-Fernandez-Blanco et al. revealed that even non-obese animals in an overweight condition (BCS 6–7) already exhibit metabolic alterations, such as greater cholesterol and insulin levels concentrations compared to lean dogs. The study identified that fasting triglyceride concentrations serve as an early marker of obesity-related metabolic dysfunction (ORMD), positively associated with markers like fructosamine and insulin.

Obesity management has evolved from simple caloric restriction to the strategic manipulation of macronutrients. The work of Godfrey et al. utilized a three-test diet approach to isolate the effects of protein, fat, and carbohydrates. The results indicated that a low-fat (LF) diet promoted greater lean soft tissue mass, while a low-carbohydrate (LC) diet showed potential benefits in satiety regulation by maintaining reduced ghrelin secretion and increasing post-prandial PYY.

Intestinal health also emerged as an important pillar of treatment. Príncipe et al. evaluated the inclusion of enzymatically hydrolyzed poultry byproduct meal (EHPM-c) in obese elderly cats. Although the diet did not alter systemic blood pressure in the short term, it promoted a beneficial modulation of the fecal microbiota. This modulation is essential for maintaining intestinal and systemic homeostasis in senior animals facing the simultaneous challenges of aging and obesity.

## Conclusion

The articles gathered in this Research Topic reinforce that the treatment of pet obesity requires a multifaceted approach. The advances presented here provide veterinarians and nutritionists with evidence-based tools to improve the research on metabolic health and longevity in companion animals. Collectively, these findings point toward a future where weight management is personalized, preventive, and focused on preserving muscle mass and intestinal health.

